# Thoracoscopic right atrial access for septal lead implantation: a novel minimally invasive technique (case report)

**DOI:** 10.1093/ehjcr/ytag319

**Published:** 2026-05-19

**Authors:** Evelyne Verhulst, Elise Bakelants, Filip Haenen

**Affiliations:** Department of Cardiac Surgery, Imelda Hospital Bonheiden, Imeldalaan 9, 2820 Bonheiden, Belgium; Department of Cardiology, Imelda Hospital Bonheiden, Imeldalaan 9, 2820 Bonheiden, Belgium; Department of Cardiac Surgery, Imelda Hospital Bonheiden, Imeldalaan 9, 2820 Bonheiden, Belgium

**Keywords:** Thoracoscopy, Right atrium, Septal pacing, Left bundle branch area pacing, Cardiac resynchronization, Minimally invasive cardiac surgery, Case report

## Abstract

**Background:**

Transvenous lead implantation remains the standard of cardiac pacing; however, alternative strategies are required in patients with limited or absent venous access. Video-assisted thoracoscopic surgery (VATS) has previously been used for epicardial left ventricular lead placement when transvenous approaches fail. Direct thoracoscopic access to the right atrium for endocardial septal pacing has not been previously described. We report a novel, minimally invasive technique allowing right atrial access and implantation of a septal pacing lead targeting the left bundle branch (LBB) area.

**Case summary:**

A 67-year-old patient with heart failure with reduced ejection fraction and LBB block was referred for cardiac resynchronization therapy. Conventional transvenous CRT implantation was unsuccessful due to bilateral subclavian venous stenosis and unsuitable coronary sinus lead positioning with high pacing thresholds and phrenic nerve stimulation. Surgical epicardial lead placement was considered high risk because of extensive intrathoracic adhesions following previous oesophageal surgery. A hybrid thoracoscopic approach was therefore pursued. Under general anaesthesia with single-lung ventilation, three 5 mm thoracoscopic ports were inserted on the right anterior thorax. After pericardial opening, a purse-string suture was placed on the right atrium, allowing introduction of a 7–9 Fr sheath using the Seldinger technique. Under fluoroscopic and electrophysiologic guidance, a pacing lead was advanced and positioned in the LBB area. Satisfactory electrical parameters were achieved. Postoperative recovery was uneventful, and device interrogation at follow-up demonstrated stable lead parameters and effective ventricular pacing.

**Discussion:**

Thoracoscopic right atrial access may represent a feasible minimally invasive strategy for septal lead implantation in patients without conventional venous access. This hybrid surgical-electrophysiological technique allows direct atrial visualization while enabling endocardial conduction system pacing. Although this initial experience demonstrates technical feasibility and procedural safety, further experience and long-term follow-up are required to determine the reproducibility and durability of this approach.

## Graphical Abstract

**Graphical Abstract:**
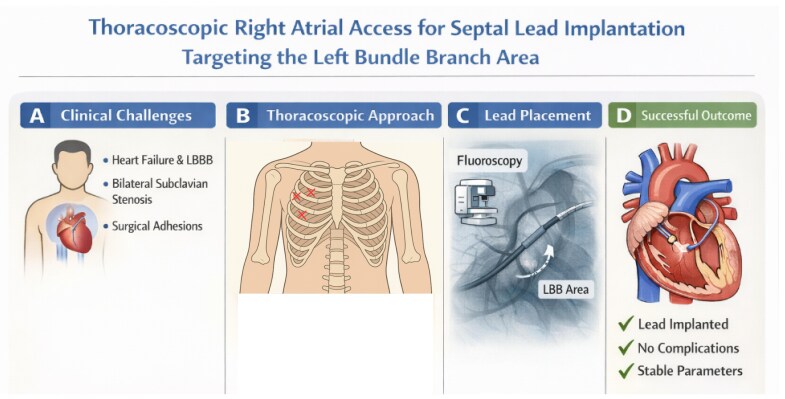

Learning points

- Cardiac resynchronization therapy (CRT) implantation may be challenging in patients with central venous obstruction or prior thoracic surgery.
- Thoracoscopic right atrial access can provide an alternative route for endocardial septal lead implantation in patients with limited venous access.
- Hybrid procedures combining minimally invasive cardiac surgery and electrophysiological lead implantation may expand therapeutic options for complex CRT implantation cases.

## Introduction

We describe a novel technique for septal lead implantation in the left bundle branch (LBB) area using thoracoscopic direct access to the right atrium. While transvenous implantation remains the standard, surgical approaches are often necessary when endovascular access is unavailable or inadequate.^1^

This novel technique is especially relevant in patients for whom traditional transvenous placement is contraindicated or unsuccessful, such as those with prior thoracic surgeries or venous occlusions. Its minimally invasive nature offers potential advantages.^1–3^ However, risks remain, including haematoma or infection at the pericardial or atrial entry site, and possible lead malposition. Fluoroscopic guidance and intraoperative mapping are essential to ensure optimal lead placement and avoid complications.^4–6^

This paper describes the technique and clinical application, emphasizing preoperative planning, surgical execution, multidisciplinary collaboration, and advanced imaging in ensuring procedural success.

## Summary figure

**Table:** 

Time point	Clinical event
Pre-procedural evaluation	A 68-year-old male with heart failure with reduced ejection fraction (LVEF ∼25%), NYHA II, and left bundle branch block (QRS 182 ms). CT imaging demonstrated bilateral stenosis of the axillary and subclavian veins.
Initial CRT-D implantation	Attempted conventional CRT implantation. Right atrial and right ventricular leads successfully implanted. Coronary sinus LV lead placement unsuccessful due to high pacing thresholds and phrenic nerve stimulation.
Intra-procedural complication	Attempt to switch to left bundle branch area pacing via transvenous access aborted due to loss of venous access.
Multidisciplinary decision	Surgical epicardial lead placement considered high risk due to extensive prior thoracic surgery and intrathoracic adhesions. Decision made to pursue thoracoscopic right atrial access for septal lead implantation.
Procedure: thoracoscopic right atrial access for septal lead implantation	Thoracoscopic access to the right atrium established. Endocardial septal lead implanted targeting the left bundle branch area.
Immediate postoperative period	Haemodynamically stable. Chest X-ray showed a small apical pneumothorax managed conservatively.
Postoperative imaging (Day 3)	Transthoracic echocardiography demonstrated severely reduced LV function without pericardial effusion and confirmed intraseptal lead positioning.
Follow-up (4 weeks)	Device interrogation demonstrated stable electrical parameters with 100% ventricular pacing and QRS narrowing from 182 to 155 ms. No arrhythmias detected.

## Case presentation

**Figure 1:**
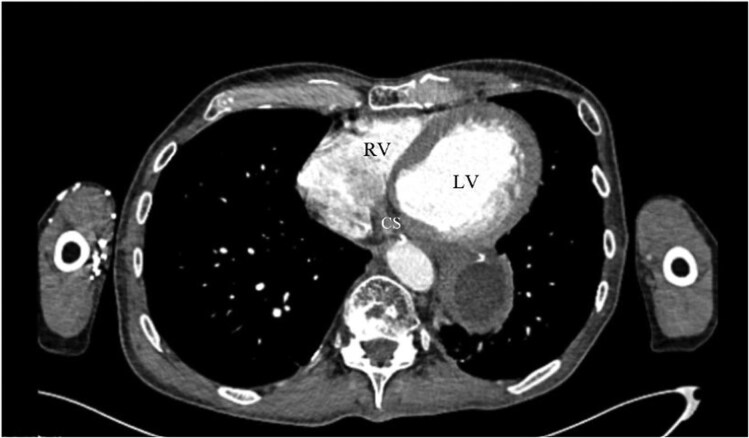
Preoperative CT showing distorted thoracic anatomy, precluding epicardial access.

**Figure 2:**
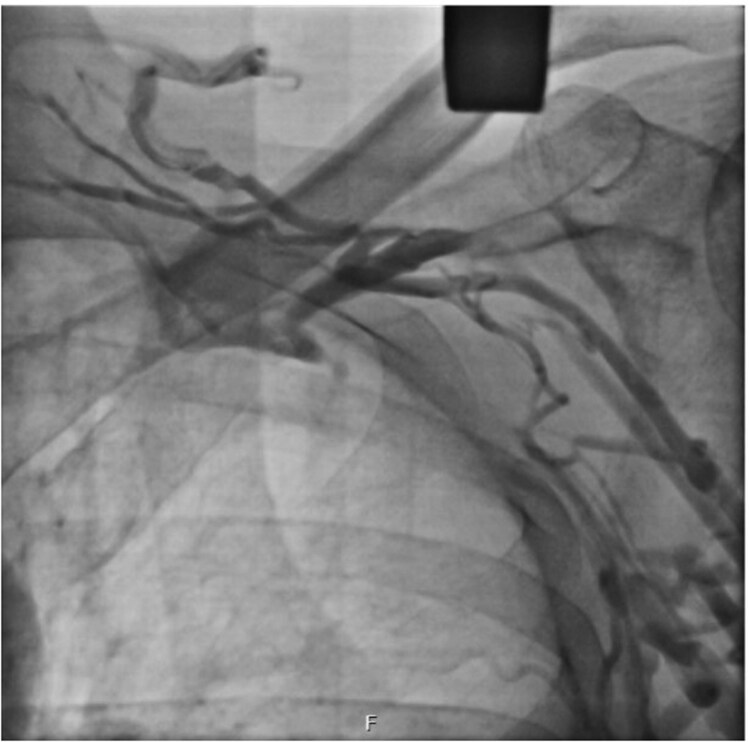
Venogram showing left-sided collateral formation consistent with subclavian vein obstruction, precluding conventional transvenous access.

A 68-year-old male with heart failure with reduced ejection fraction (HFrEF) was referred for cardiac resynchronization therapy (CRT). Baseline clinical characteristics, electrocardiographic findings, and echocardiographic parameters are summarized in *Table 1*. The patient had a history of left-sided thoracic surgeries, including subtotal oesophagectomy and partial gastrectomy for oesophageal adenocarcinoma, resulting in significant adhesions and distorted anatomy (*Figure 1*: CT showing hostile thorax). The preoperative thoracic CT was performed to evaluate the feasibility of thoracoscopic access and assess venous anatomy. Imaging demonstrated bilateral stenosis of the axillary and subclavian veins, significantly limiting transvenous access. This was confirmed by venography, showing extensive collateral formation (*Figure 2*). There was no history of device-related infection, and the patient was not receiving anticoagulation therapy.

**Table 1:** Baseline clinical, electrocardiographic, and echocardiographic characteristics

Parameter	Value
Age	68 years
Sex	Male
Heart failure classification	NYHA Class II
Heart rhythm	Sinus rhythm
Heart rate	62 b.p.m.
QRS morphology	Left bundle branch block
QRS duration	182 ms
PR interval	186 ms
QTc	493 ms
Left ventricular ejection fraction	25%
LV function	Diffuse hypokinesia
LV morphology	Mild dilatation
Right ventricular function	Preserved (TAPSE ≥20 mm)

During the initial implantation, balloon dilatation of the right subclavian vein was performed to address venous obstruction. A right atrial lead and right ventricular defibrillation lead were successfully implanted. A quadripolar left ventricular lead was placed in an anterolateral coronary sinus tributary, but pacing thresholds were high and phrenic nerve stimulation occurred, rendering this site unsuitable. The procedure was therefore converted to an attempt at LBB area pacing; however, venous access was lost, and the procedure was abandoned. Due to elevated surgical risks, open epicardial lead placement was considered contraindicated. Therefore, thoracoscopic direct right atrial access was selected as an innovative alternative for the implantation of a pacing lead targeting the LBB area.

The procedure was performed under general anaesthesia with the patient in the dorsal decubitus position, using single-lung ventilation. Three 5 mm trocars were placed in the third, fourth, and fifth intercostal spaces on the anterior thorax.

After careful dissection and opening of the pericardium, suspension sutures were placed to maintain exposure. A purse-string barbed suture was positioned on the right atrium, and a 7 Fr sheath was introduced parasternally using the Seldinger technique, into the right atrium, under direct vision. The procedure was performed using a stylet-driven pacing lead (Biotronik Solia S60). The optimal implantation site was determined in collaboration with the electrophysiology team.

**Figure 3:**
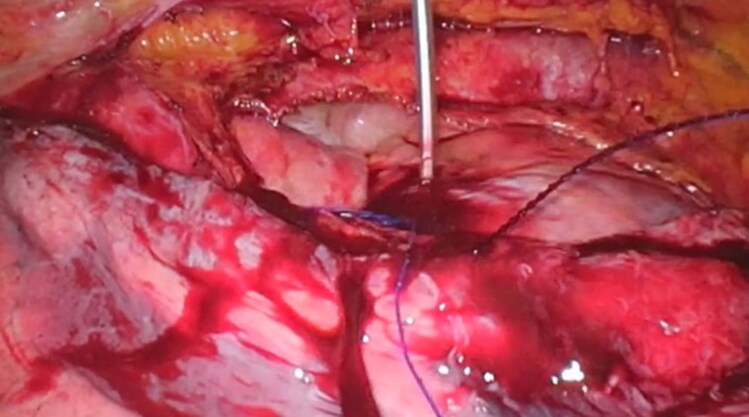
The lead was secured with appropriate slack and connected to the new device. A purse-string suture was then tightened.

Lead positioning is the most technically demanding aspect. Multiple attempts were required to obtain an adequate position. Several positions demonstrated high capture thresholds or mechanical resistance to lead advancement. The final lead position was achieved in a low septal location. Electrical parameters demonstrated a left ventricular activation time (LVAT) of 70 ms and an RV1–V6 interpeak interval of 40 ms. Although classical criteria for selective LBB capture were not fully met, they were considered acceptable given the extensive septal fibrosis. Final pacing thresholds improved to <1 V in both unipolar and bipolar configurations. The lead was then secured, slack adjusted, and connected to the new device. The purse-string suture was secured, and the incisions were closed in layers, leaving a thoracic drain in place (*Figure 3*).

An intraoperative video demonstrating the procedural steps is available (Supplementary material online, *Video S1*).

No therapeutic anticoagulation was administered intraoperatively. Postoperatively, prophylactic anticoagulation with nadroparin (Fraxiparine® 3800 IU daily) was initiated for venous thromboembolism prevention, and low-dose acetylsalicylic acid (100 mg daily) was started as antiplatelet therapy.

The postoperative course was uneventful. The patient remained haemodynamically stable, and postoperative transthoracic echocardiography on Day 3 demonstrated no pericardial effusion and clear visualization of the intraseptal pacing lead. The thoracic drain was removed on Day 2 postoperatively. Chest X-ray confirmed correct lead positioning and revealed a small apical pneumothorax, which was managed conservatively. At 4-week follow-up, device interrogation demonstrated stable lead parameters and normal device function. Electrical measurements are summarized in *Table 2*. Device interrogation showed 100% biventricular pacing without recorded arrhythmias. The patient remained clinically stable without heart failure hospitalizations.

**Table 2:** Device parameters at 4-week follow-up

Lead	Capture threshold	Sensing	Impedance
Atrial lead	0.75 V at 0.4 ms	0.5 mV	400 Ω
Right ventricular lead	0.75 V at 0.4 ms	6.5 mV	361 Ω
Left bundle branch area lead	1.25 V at 0.4 ms	—	646 Ω

## Discussion

CRT is an established treatment for patients with HFrEF and left bundle branch block, improving symptoms, ventricular function, and survival. Conventional CRT relies on transvenous placement of a left ventricular lead through the coronary sinus. However, this approach may be unsuccessful due to unfavourable venous anatomy, phrenic nerve stimulation, or high pacing thresholds. In such circumstances, alternative pacing strategies must be considered.

In the present case, several factors limited conventional CRT implementation. The patient exhibited bilateral stenosis of the axillary and subclavian veins, restricting reliable transvenous access. Although a coronary sinus lead was successfully introduced, it demonstrated high pacing thresholds and phrenic nerve stimulation, making this location unsuitable for chronic pacing. Furthermore, extensive prior thoracic surgery resulted in significant adhesions and distorted anatomy, rendering surgical epicardial lead placement via thoracotomy high risk.

Thoracoscopic right atrial access was used to facilitate endocardial septal lead implantation targeting the LBB area. To our knowledge, this technique has not been previously described for this indication. Video-assisted thoracoscopic surgery (VATS) has been employed to place an epicardial LV lead when transvenous approaches fail, demonstrating the feasibility of minimally invasive surgical access for pacing procedures.^5,6^

This approach is particularly advantageous for patients in whom standard transvenous lead implantation is not feasible or entails significant risk. Ideal candidates include:

- Patients with venous anomalies or obstructions, such as congenital anomalies or venous thrombosis, may encounter challenges.^7–9^
- Patients with prior thoracic surgery and adhesions making epicardial access via thoracotomy high risk.^7–9^

The described approach offers a valuable alternative in selected patients where conventional or surgical methods are not feasible. Importantly, it should not be viewed as superior to standard right ventricular lead implantation, but rather as a targeted solution for anatomically complex or high-risk scenarios. In patients with prior thoracic surgeries, altered venous anatomy, or prosthetic valves, traditional routes may carry an elevated risk or be technically unachievable.^10,11^ Compared to open thoracotomy for epicardial LV lead placement, the minimally invasive approach may lead to reduced operative trauma and shorter hospital stays.^10,12^

Several other alternative strategies include venoplasty of occluded central veins, femoral or iliac venous access for device implantation, inside-out venous access techniques, and surgical epicardial lead placement. Each option has specific limitations, including technical complexity, lead instability, or increased infection risk.

Careful preoperative evaluation is essential. Advanced imaging allows identification of venous obstruction and assessment of thoracic anatomy following previous surgery, facilitating procedural planning. Intraoperative electrophysiological guidance is essential when targeting the LBB area, as accurate depth and positioning are critical to achieve physiologic pacing.

Potential complications include bleeding at the atrial puncture site, pericardial effusion, pneumothorax, and thromboembolic events. In the present case, bleeding risk was minimized by placement of a purse-string suture prior to sheath insertion, allowing secure haemostasis. Postoperative echocardiography confirmed the absence of pericardial effusion and stable lead positioning. Careful perioperative monitoring and multidisciplinary collaboration between cardiothoracic surgeons and electrophysiologists were essential to ensure procedural safety.

Several limitations should be acknowledged. First, this report describes a single case, and the reproducibility requires further evaluation in larger patient series. Second, the procedure necessitates general anaesthesia with one-lung ventilation, which may pose risks in patients with compromised ventricular function. Third, thoracoscopic atrial access demands advanced surgical expertise in minimally invasive cardiac techniques.

## Conclusion

This novel technique represents a minimally invasive alternative for septal lead implantation in select patients who may not be suitable candidates for conventional transvenous methods. The procedure underscores the importance of a multidisciplinary approach, advanced imaging, and surgical expertise.

Although promising, further experience and longer-term follow-up are required to define its role within the spectrum of alternative CRT implantation strategies.

## Supplementary Material

ytag319_Supplementary_Data

## Data Availability

The data underlying this article are available in the article and in its online Supplementary material.

## Lead author biography

Dr. Elise Bakelants is a cardiologist and cardiac electrophysiologist at Imelda Hospital Bonheiden, Belgium, specializing in device therapy and advanced cardiac pacing. Their clinical focus includes complex pacemaker and defibrillator implantation and cardiac resynchronization therapy, with a particular interest in innovative techniques for patients with challenging anatomy or limited venous access. The procedure described in this case was performed in collaboration with Dr. Filip Haenen, a cardiothoracic surgeon specializing in minimally invasive approaches, and Dr. Evelyne Verhulst, a surgical resident and PhD candidate.

## Notes

**Consent:** Written informed consent was obtained from the patient for publication of this case report, including all associated clinical images, procedural details, and any Supplementary material. The patient was informed that anonymized data would be presented and that personal identifiers would not be disclosed.
